# 263. A preliminary analysis of vaginal microbial changes prior to incident bacterial vaginosis as determined by qPCR and inferred absolute abundance

**DOI:** 10.1093/ofid/ofad500.335

**Published:** 2023-11-27

**Authors:** Jacob H Elnaggar, Christopher M Taylor, Caleb M Ardizzone, Kristal J Aaron, Isaac C Eastlund, Keonte J Graves, Meng Luo, Alison J Quayle, Nuno Cerca, Christina A Muzny

**Affiliations:** Louisiana State University Health Sciences Center, New Orleans, Louisiana; Louisiana State University Health Sciences Center, New Orleans, Louisiana; Louisiana State University Health Sciences Center, New Orleans, Louisiana; University of Alabama at Birmingham, Birmingham, Alabama; University of Alabama at Birmingham, Birmingham, Alabama; University of Alabama at Birmingham, Birmingham, Alabama; Louisiana State University Health Sciences Center, New Orleans, Louisiana; Louisiana State University Health Sciences Center, New Orleans, Louisiana; Minho University, Braga, Braga, Portugal; University of Alabama at Birmingham, Birmingham, Alabama

## Abstract

**Background:**

Bacterial vaginosis (BV) is the most common vaginal infection among reproductive-aged women worldwide. It is associated with multiple adverse obstetric and gynecologic outcomes, yet its etiology remains unknown. BV is characterized by a decrease in protective lactobacilli (e.g., *Lactobacillus crispatus*), and a substantial increase in BV-associated bacteria (BVAB), particularly *Gardnerella vaginalis*. We investigated the vaginal microbiota to identify changes prior to, during, and immediately after incident BV (iBV).

**Methods:**

Non-pregnant sexually active women who have sex with men ages 18-45 with normal baseline vaginal microbiota (no Amsel criteria, Nugent score 0-3) were enrolled and followed for iBV using twice-daily self-collected vaginal specimens. 16S rRNA gene sequencing was performed on specimens collected during the 14 days prior to BV (pre-BV), during BV (Nugent score 7-10), and days post-BV among cases. A similar number of specimens from age, race, and menstrual cycle day matched controls maintaining normal vaginal microbiota were also sequenced. qPCR was also performed on all specimens to determine total bacterial burden and inferred absolute abundance (IAA) of key vaginal bacteria, particularly *G. vaginalis*, *L. crispatus,* and *L. iners*.

**Results:**

Vaginal specimens from 5 BV cases (n=169) and 5 matched controls (n=169; pre-BV [n=121], during BV [n=32], and post-BV [n=15]) were analyzed. Overall, a significantly higher bacterial burden was observed in specimens from BV cases compared to matched controls (Fig 1A). In BV cases, specimens collected on the day of BV had a significantly lower bacterial burden than specimens collected pre- and post-BV (Fig 1B). When comparing changes in the IAA of our vaginal bacteria of interest, we observed significantly higher amounts of *G. vaginalis* during BV (Fig 1C). There was a significant decrease in the IAA of *L. crispatus* during BV (Fig 1D), whereas *L. iners* IAA increased in post-BV (Fig 1E).

Comparing qPCR and inferred absolute abundance (IAA) between vaginal specimens collected from women with incident BV (cases) and healthy controls maintaining normal vaginal microbiota.

**Figure:**
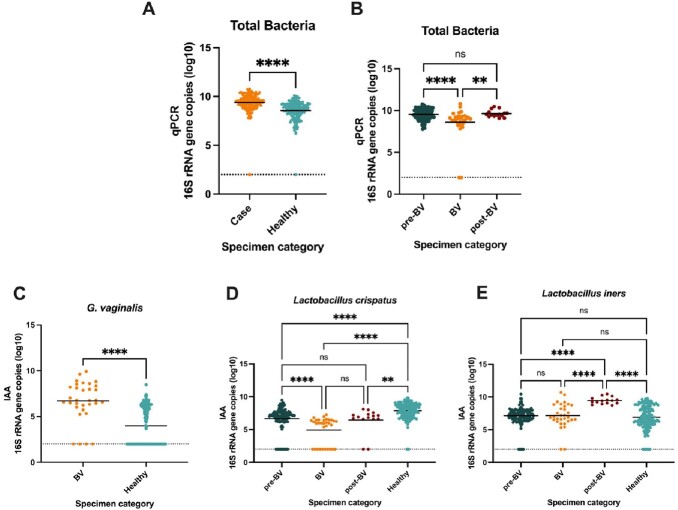

A, B. qPCR derived total bacterial burden between vaginal specimens from incident BV cases and healthy controls. C- E. inferred absolute abundance (IAA) of G. vaginalis, L. crispatus, and L. iners between vaginal specimens from incident BV cases and healthy controls. A, C. Mann Whitney test; B, D, E. Kruskal-Wallis test *p≤ 0.05, **p≤ 0.01, ***p≤ 0.001, ****p≤ 0.0001.

**Conclusion:**

These preliminary results support the hypothesis that a sequence of changes in the abundance of key vaginal bacteria occur prior to BV. During BV, we observed an increase in *G. vaginalis* while protective *L. crispatus* decreased, and *L iners* increased after BV. We anticipate expanding this analysis to include additional key BVAB.

**Disclosures:**

**Christina A. Muzny, MD, MSPH**, Abbott Molecular: Grant/Research Support|Abbott Molecular: Honoraria|BioNTech: Advisor/Consultant|Cepheid: Advisor/Consultant|Cepheid: Honoraria|Elsevier: Honoraria|Gilead: Grant/Research Support|Lupin Pharmaceuticals: Grant/Research Support|Roche: Honoraria|Scynexis: Advisor/Consultant|Scynexis: Honoraria|Visby Medical: Honoraria

